# Supplementary material for: Maternal Exposure to Ambient Air Pollution and Risk of Preeclampsia: A Population-Based Cohort Study in Scania, Sweden
Source: Int J Environ Res Public Health. 2020 Mar 7;17(5):1744. doi: 10.3390/ijerph17051744 (PMC7084298; doi:10.3390/ijerph17051744)
Supplement: Supplementary file 1 [file ijerph-17-01744-s001.pdf]

International Journal of
Environmental Research and Public Health

MDPI

Supplementary materials

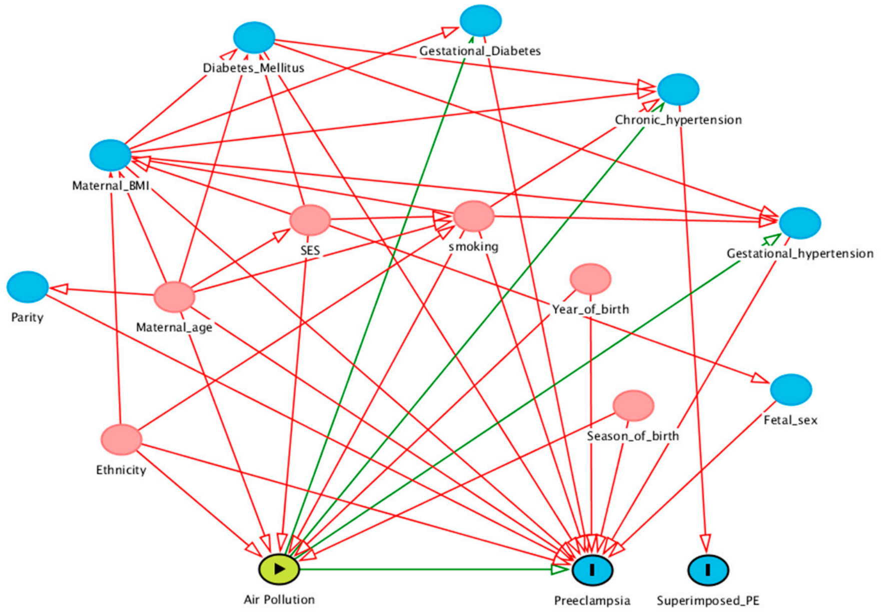

Figure S1. Directed Acyclic Graphs to guide the selection of potential confounders for the association between the ambient air pollution and preeclampsia: (yellow) exposure; (blue with l) outcome; (red dot) common cause; (blue dot) ancestor.

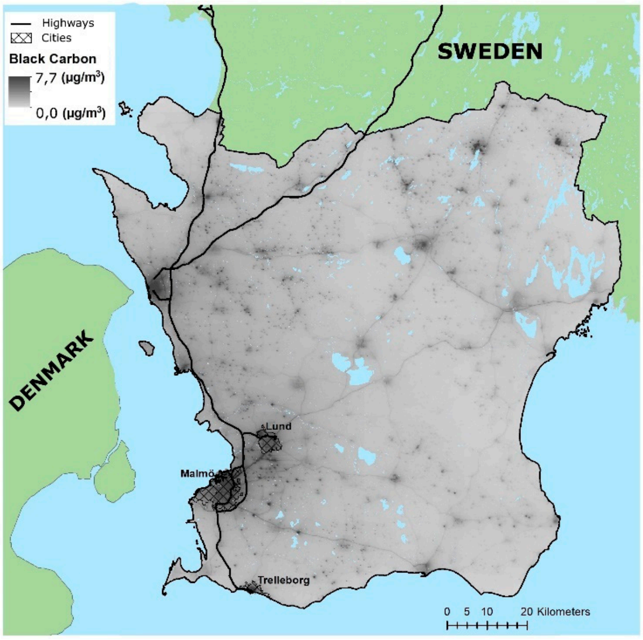

Figure S2. Local black carbon (BC) dispersion modeling in Scania, Sweden in January 2006. Map by Emilie Stroh.

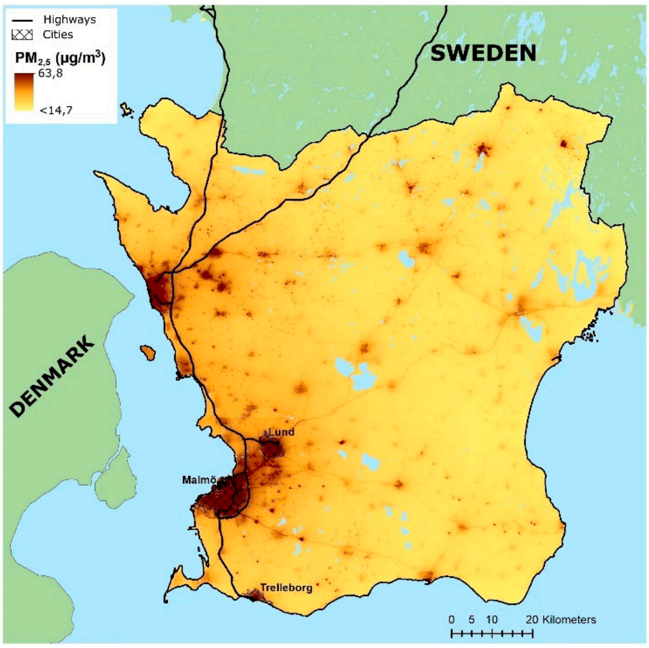

Figure S3. Local $PM_{2.5}$ dispersion modeling in Scania, Sweden in January 2006. Map by Emilie Stroh.

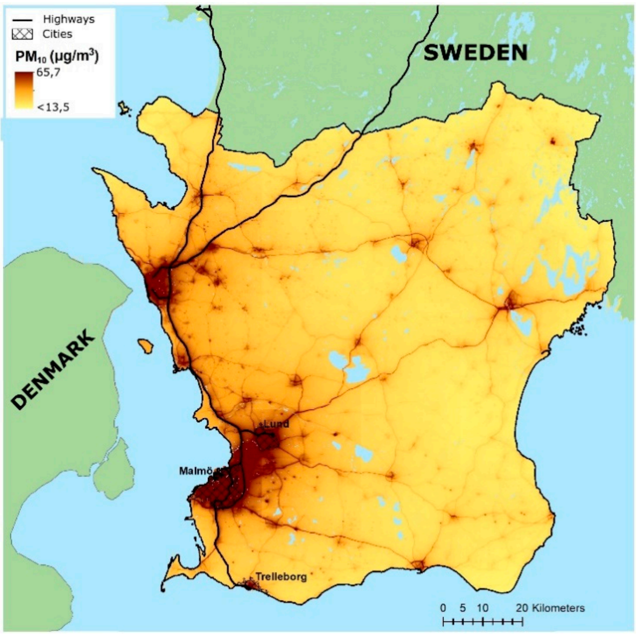

Figure S4. Local PM_{10} dispersion modeling in Scania, Sweden in January 2006. Map by Emilie Stroh.

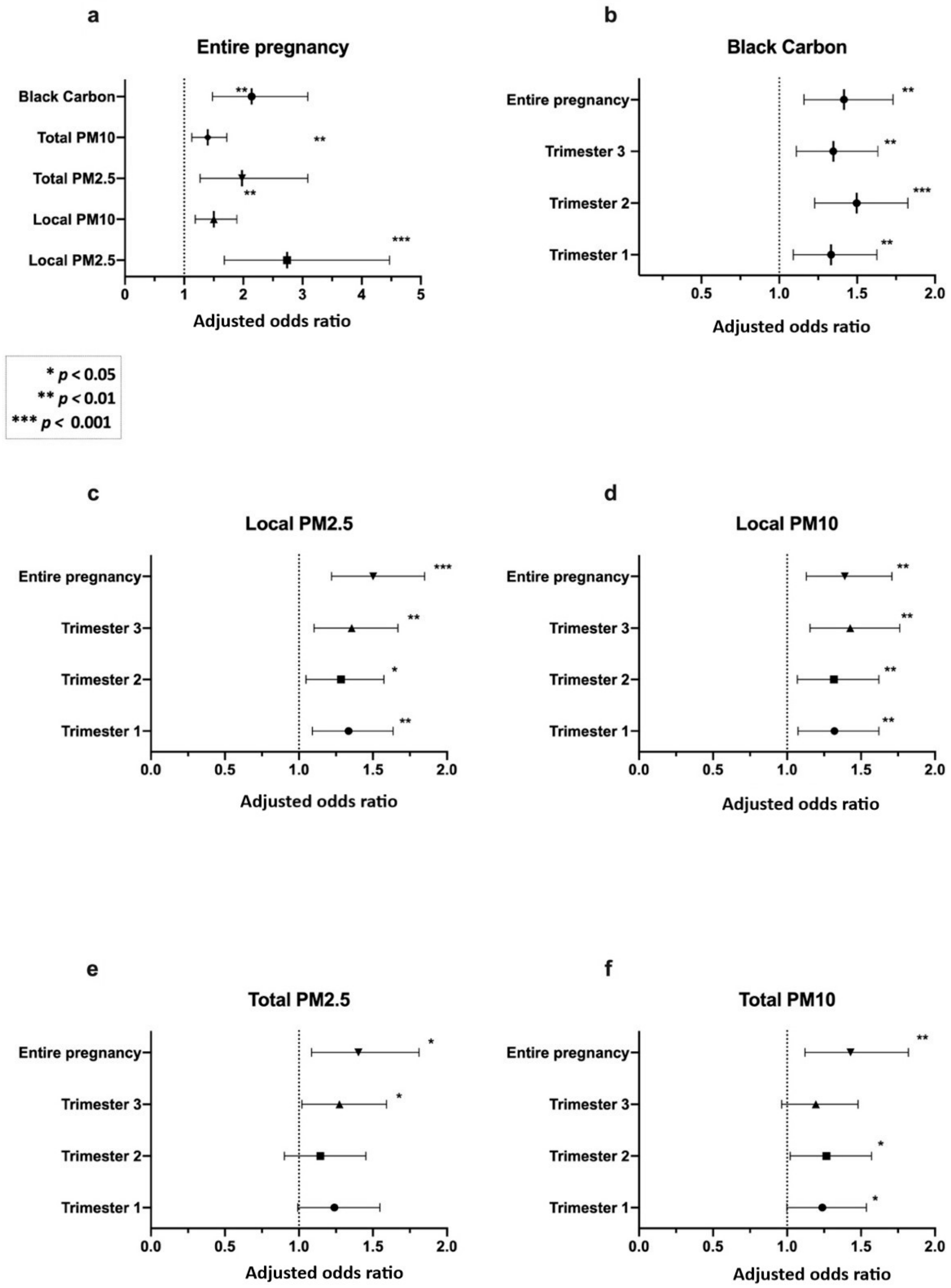

Figure S5. Graphic illustration of adjusted analysis of all particles. (a) Linear exposure trend in whole pregnancy. (b) Adjusted Odds Ratios (AORs) for PE at the highest quartiles of black carbon exposure in all exposure windows. (c) AORs for PE at the highest quartiles of local $PM_{2.5}$ exposure in all exposure windows. (d) AORs for PE at the highest quartiles of local PM_{10} exposure in all exposure windows. (e) AORs for PE at the highest quartiles of total $PM_{2.5}$ exposure in all exposure windows. (f) AORs for PE at the highest quartiles of total PM_{10} exposure in all exposure windows. p-values for (a) as compared to the healthy controls and for (b), (c), (d), (e) and (f) as compared to the lowest quartiles in each specific exposure windows.

Table S1. Pearson's correlations among pollutants (μg/m^3) in Scania, Sweden over the 2000–2009 period.

	NO_x	Local $PM_{2.5}$	Local PM_{10}	BC	Total $PM_{2.5}$	Total PM_{10}
**Entire pregnancy**						
NO_x	1.00					
Local $PM_{2.5}$	0.90**	1.00				
Local PM_{10}	0.86**	0.96*	1.00			
BC	0.85**	0.87**	0.85**	1.00		
Total $PM_{2.5}$	0.58**	0.70**	0.69**	0.56**	1.00	
Total PM_{10}	0.62**	0.63**	0.62**	0.55**	0.75**	1.00
**First trimester**						
NO_x	1.00					
Local $PM_{2.5}$	0.81**	1.00				
Local PM_{10}	0.73**	0.94**	1.00			
BC	0.74**	0.77**	0.70**	1.00		
Total $PM_{2.5}$	0.37**	0.56**	0.56**	0.35**	1.00	
Total PM_{10}	0.40**	0.42**	0.43**	0.37**	0.73**	1.00
**Second trimester**						
NO_x	1.00					
Local $PM_{2.5}$	0.82**	1.00				
Local PM_{10}	0.72**	0.94**	1.00			
BC	0.81**	0.69**	0.61**	1.00		
Total $PM_{2.5}$	0.37**	0.60**	0.61**	0.26**	1.00	
Total PM_{10}	0.41**	0.46**	0.45**	0.34**	0.74**	1.00
**Third trimester**						
NO_x	1.00					
Local $PM_{2.5}$	0.79**	1.00				
Local PM_{10}	0.73**	0.94**	1.00			
BC	0.78**	0.72**	0.67**	1.00		
Total $PM_{2.5}$	0.37**	0.53**	0.55**	0.32**	1.00	
Total PM_{10}	0.40**	0.42**	0.42**	0.38**	0.74**	1.00

** Correlation is significant at the 0.01 level (2-tailed).

Table S2. Confounder characteristics among the second trimester-specific black carbon exposure quartiles (n = 33,475) over the 2000–2009 period.

Characteristics	First quartile *n* (%)	Second quartile *n* (%)	Third quartile *n* (%)	Fourth quartile *n* (%)
Births	8,303	8,666	7,983	8,523
Maternal age				
≤19	104 (1.3)	87 (1.0)	104 (1.3)	123 (1.4)
20-34	6,376 (76.8)	6,829 (78.8)	6,343 (79.5)	7,031 (82.5)
≥35	1,823 (22.0)	1,750 (20.2)	1,536 (19.2)	1,369 (16.1)
Parity				
Nulliparous	3,433 (41.3)	3,944 (45.5)	4,165 (52.2)	4,552 (53.4)
Parous	4,870 (58.7)	4,722 (54.5)	3,818 (47.8)	3,971 (46.6)

Pre-pregnancy BMI*				
<18.5	164 (2.0)	237 (2.7)	217 (2.7)	230 (2.7)
18.5-24.9	4,989 (60.1)	5,455 (62.9)	5,210 (65.3)	5,265 (68.1)
25-29.9	2,171 (26.1)	2,105 (24.3)	1,851 (23.2)	2,104 (24.7)
≥30	979 (11.8)	869 (10.0)	705 (8.8)	924 (10.8)
Smoking†				
Non-smoker	7,537 (90.8)	7,853 (90.6)	7,305 (91.5)	7,513 (88.1)
<10	548 (6.6)	610 (7.0)	481 (6.0)	687 (8.1)
≥10	218 (2.6)	203 (2.3)	197 (2.5)	323 (3.8)
Maternal health				
Diabetes Mellitus	58 (0.7)	44 (0.5)	62 (0.8)	64 (0.8)
Gestational diabetes	172 (2.1)	199 (2.3)	241 (3.0)	288 (3.4)
Essential hypertension	41 (0.5)	46 (0.5)	27 (0.3)	53 (0.6)
Gestational hypertension	183 (2.2)	146 (1.7)	118 (1.5)	106 (1.2)
Maternal education				
Pre-secondary	665 (8.0)	889 (10.3)	1,129 (14.1)	1,641 (19.3)
Secondary	4,129 (49.7)	3,706 (42.8)	3,024 (37.9)	3,704 (43.5)
Post-secondary	3,509 (42.3)	4,071 (47.0)	3,830 (48.0)	3,178 (37.3)
Household income‡				
<200,000	903 (10.9)	1,366 (15.8)	2,115 (26.5)	2,792 (32.8)
200,000-300,000	1,504 (18.1)	1,802 (20.8)	2,002 (25.1)	2,730 (32.0)
300,000-400,000	2,804 (33.8)	2,761 (31.9)	2,096 (26.3)	1,952 (22.9)
>400,000	3,092 (37.2)	2,737 (31.6)	1,770 (22.2)	1,049 (12.3)
Maternal country of birth				
Nordic	7,472 (90.0)	6,885 (79.4)	5,293 (66.3)	5,096 (59.8)
Other	831 (10.0)	1,781 (20.6)	2,690 (33.7)	3,427 (40.2)
Offspring sex				
Male	4,275 (51.5)	4,389 (50.6)	4,096 (51.3)	4,383 (51.4)
Female	4,028 (48.5)	4,277 (49.4)	3,887 (48.7)	4,140 (48.6)
Year of birth				
2000	277 (3.3)	323 (3.7)	302 (3.8)	455 (5.3)
2001	696 (8.4)	861 (9.9)	745 (9.3)	880 (10.3)
2002	728 (8.8)	846 (9.8)	816 (10.2)	1,066 (12.5)
2003	859 (10.3)	920 (10.6)	838 (10.5)	1,111 (13.0)
2004	758 (9.1)	929 (10.7)	821 (10.3)	1,328 (15.6)
2005	888 (10.7)	824 (9.5)	920 (11.5)	1,068 (12.5)
2006	806 (9.7)	808 (9.3)	934 (11.7)	997 (11.7)
2007	952 (11.5)	1,103 (12.7)	960 (12.0)	968 (11.4)
2008	1,518 (18.3)	1,311 (15.1)	882 (11.0)	231 (2.7)
2009	821 (9.9)	741 (8.6)	765 (9.6)	419 (4.9)
Season of birth				
Winter	1,794 (21.6)	1,927 (22.2)	1,600 (20.0)	2,223 (26.1)
Spring	1,952 (23.5)	2,198 (25.4)	1,852 (23.2)	2,561 (30.0)
Summer	2,258 (27.2)	2,251 (26.0)	2,244 (28.1)	2,187 (25.7)
Autumn	2,299 (27.7)	2,290 (26.4)	2,287 (28.6)	1,552 (18.2)

* Body Mass Index; † Cigarettes per day; ‡ Measured in Swedish kronor (SEK) per year.

Table S3. Outcome events for total preeclampsia in relation to the lowest and highest exposure quartiles of all pollutants during each window of exposure.

Pollutant	Exposure window	Non-PE, n (%)	PE, n (%)	Total, n (%)	Lowest exposure quartile, n (%)	Highest exposure quartile, n (%)
Black carbon	Entire pregnancy	31,406 (97.1)	935 (2.9)	32,341 (90.9)	203 (21.7)	279 (29.8)**
	1st trimester	32,574 (97.1)	970 (2.9)	33,544 (94.3)	219 (22.6)	283 (29.2)**
	2nd trimester	32,499 (97.1)	976 (2.9)	33,475 (94.1)	211 (21.6)	302 (30.9)**
	3rd trimester	32,624 (97.1)	982 (2.9)	33,606 (94.5)	223 (22.7)	304 (31.0)**

Local $PM_{2.5}$	Entire pregnancy	29,999 (97.1)	893 (2.9)	30,892 (86.8)	195 (21.8)	280 (31.4)**
	1st trimester	32,124 (97.1)	962 (2.9)	33,086 (93.0)	232 (24.1)	268 (27.9) *
	2nd trimester	31,601 (97.1)	946 (2.9)	32,547 (91.5)	222 (23.5)	285 (30.1)**
	3rd trimester	32,594 (97.1)	979 (2.9)	33,573 (94.4)	206 (21.0)	283 (28.9)**
Local PM_{10}	Entire pregnancy	30,125 (97.1)	908 (2.9)	31,033 (87.2)	203 (22.4)	276 (30.4)**
	1st trimester	32,427 (97.1)	973 (2.9)	33,400 (93.9)	235 (24.2)	263 (27.0)
	2nd trimester	31,656 (97.1)	954 (2.9)	32,610 (91.7)	218 (22.9)	284 (29.8)**
	3rd trimester	32,268 (97.1)	972 (2.9)	33,240 (93.4)	201 (20.7)	293 (30.1)**
Total $PM_{2.5}$	Entire pregnancy	24,308 (97.0)	742 (3.0)	25,050 (70.4)	166 (22.4)	205 (27.6)
	1st trimester	30,194 (97.1)	909 (2.9)	31,103 (87.4)	214 (23.5)	220 (24.2)
	2nd trimester	27,624 (97.0)	844 (3.0)	28,468 (80.0)	185 (21.9)	216 (25.6)
	3rd trimester	26,832 (97.0)	827 (3.0)	27,659 (77.8)	191 (23.1)	252 (30.5)**
Total PM_{10}	Entire pregnancy	30,130 (97.1)	909 (2.9)	31,039 (87.3)	223 (24.5)	249 (27.4)
	1st trimester	32,437 (97.1)	974 (2.9)	33,411 (93.9)	211 (21.7)	248 (25.5)*
	2nd trimester	31,651 (97.1)	954 (2.9)	32,605 (91.7)	230 (24.1)	255 (26.7)
	3rd trimester	32,268 (97.1)	972 (2.9)	33,240 (93.4)	239 (24.6)	263 (27.1)
NO_x	Entire pregnancy	32,323 (97.1)	974 (2.9)	33,297 (93.6)	211 (21.7)	271 (27.8)**
	1st trimester	32,832 (97.1)	984 (2.9)	33,816 (95.1)	228 (23.2)	271 (27.5)**
	2nd trimester	33,168 (97.1)	1,002 (2.9)	34,170 (96.1)	223 (22.3)	276 (27.5)**
	3rd trimester	34,415 (97.1)	1,031 (2.9)	35,446 (99.7)	218 (21.1)	299 (29.0)**

*p <0.05 (compared with the lowest quartile). ** p <0.01 (compared with the lowest quartile).

Table S4. Adjusted odds ratios (AOR) from the complete case analysis (CCA) on the effects of maternal exposure to ambient particles on risk for preeclampsia associated with a 1 μg/m³ increase in the concentration of BC and 5 μg/m³ increase in the concentration of local and total $PM_{2.5}$ and PM_{10} (with the intermediate variables[a] derived from DAGs).

Exposure	Entire pregnancy AOR§ (95% CI)	First trimester AOR§ (95% CI)	Second trimester AOR§ (95% CI)	Third trimester AOR§ (95% CI)
**Black carbon† (n/N)**	935/32,341	970/33,544	976/33,475	982/33,606
	2.14** (1.48, 3.09)	1.89** (1.34, 2.65)	2.08** (1.45, 2.98)	1.77** (1.28, 2.45)
**Local $PM_{2.5}$ ‡ (n/N)**	893/30,892	962/33,086	946/32,547	979/33,573
	2.74** (1.68, 4.47)	2.01** (1.34, 3.02)	2.11** (1.42, 3.13)	1.78** (1.22, 2.61)
**Local PM_{10} ‡ (n/N)**	908/31,033	973/33,400	954/32,610	972/33,240
	1.50** (1.19, 1.89)	1.31** (1.09, 1.58)	1.32** (1.11, 1.58)	1.32** (1.11, 1.57)
**Total $PM_{2.5}$ ‡ (n/N)**	742/25,050	909/31,103	844/28,468	827/27,659
	1.98** (1.27, 3.09)	1.31* (1.06, 1.61)	1.14 (0.92, 1.42)	1.27* (1.03, 1.56)
**Total PM_{10} ‡ (n/N)**	909/31,039	974/33,411	954/32,605	972/33,240
	1.40** (1.13, 1.72)	1.24** (1.09, 1.41)	1.10 (0.97, 1.26)	1.11 (0.98, 1.24)

[a]Intermediate variables are gestational diabetes, essential hypertension, gestational hypertension. †Estimates are for each 1 μg/m³ increase of black carbon particles. ‡Estimates are for each 5 μg/m³ increase of local and total $PM_{2.5}$ and PM_{10}. §Adjusted for maternal age, body mass index, parity, smoking, diabetes mellitus, gestational diabetes, essential hypertension, gestational hypertension, maternal country of birth, education level, annual household income, fetal sex, year and season of

birth. *p-value <0.05 as compared to the healthy controls. **p-value <0.01 as compared to the healthy controls.

Table S5. Adjusted odds ratios (AOR) from the complete case analysis (CCA) on the effects of maternal exposure to ambient particles on risk for preeclampsia associated with a 1 μg/m³ increase in the concentration of BC and 5 μg/m³ increase in the concentration of local and total $PM_{2.5}$ and PM_{10} (without the intermediate variables[a] derived from DAGs).

Exposure	Entire pregnancy AOR[§] (95% CI)	First trimester AOR[§] (95% CI)	Second trimester AOR[§] (95% CI)	Third trimester AOR[§] (95% CI)
**Black carbon[†] (n/N)**	935/32,341	970/33,544	976/33,475	982/33,606
	2.00** (1.39, 2.88)	1.83** (1.31, 2.56)	1.97** (1.38, 2.81)	1.71** (1.24, 2.35)
**Local $PM_{2.5}$ [‡] (n/N)**	893/30,892	962/33,086	946/32,547	979/33,573
	2.74** (1.69, 4.44)	2.00** (1.34, 2.99)	2.09** (1.42, 3.08)	1.76** (1.21, 2.56)
**Local PM_{10} [‡] (n/N)**	908/31,033	973/33,400	954/32,610	972/33,240
	1.48** (1.18, 1.87)	1.31* (1.09, 1.57)	1.31** (1.10, 1.56)	1.31** (1.10, 1.56)
**Total $PM_{2.5}$ [‡] (n/N)**	742/25,050	909/31,103	844/28,468	827/27,659
	2.09** (1.35, 3.24)	1.30* (1.06, 1.59)	1.16 (0.94, 1.43)	1.27* (1.04, 1.56)
**Total PM_{10} [‡] (n/N)**	909/31,039	974/33,411	954/32,605	972/33,240
	1.40** (1.14, 1.72)	1.23** (1.08, 1.40)	1.11 (0.98, 1.26)	1.11 (0.98, 1.24)

[a]Intermediate variables are gestational diabetes, essential hypertension, gestational hypertension. [†]Estimates are for each 1 μg/m³ increase of black carbon particles. [‡]Estimates are for each 5 μg/m³ increase of local and total $PM_{2.5}$ and PM_{10}. [§]Adjusted for maternal age, body mass index, parity, smoking, diabetes mellitus, maternal country of birth, education level, annual household income, fetal sex, year and season of birth. *p-value <0.05 as compared to the healthy controls. **p-value <0.01 as compared to the healthy controls.

Table S6. Adjusted analysis for late-onset preeclampsia in relation to the quartile-specific exposure of all pollutants during each exposure window over the 2000-2009 period (n=43,688).

Pollutant	Exposure window	Non-PE, n	Late-onset PE, n	Quartile 2 AOR[§] (95% CI)	Quartile 3 AOR[§] (95% CI)	Quartile 4 AOR[§] (95% CI)
**Black carbon**	Entire pregnancy	38,124	1,129	1.07 (0.90, 1.28)	1.16 (0.97, 1.39)	1.22* (1.02, 1.46)
	1st trimester	39,648	1,175	0.98 (0.82, 1.17)	1.13 (0.95, 1.35)	1.14 (0.95, 1.37)
	2nd trimester	39,607	1,178	1.13 (0.95, 1.34)	1.11 (0.93, 1.34)	1.28** (1.07, 1.54)
	3rd trimester	39,853	1,184	0.96 (0.81, 1.15)	1.06 (0.89, 1.27)	1.19* (1.00, 1.42)
**Local $PM_{2.5}$**	Entire pregnancy	36,430	1,076	1.05 (0.87, 1.26)	1.13 (0.94, 1.36)	1.31** (1.08, 1.58)
	1st trimester	39,088	1,160	0.94 (0.79, 1.12)	1.09 (0.92, 1.30)	1.15 (0.96, 1.38)
	2nd trimester	38,530	1,141	1.01 (0.85, 1.21)	0.98 (0.82, 1.18)	1.19 (0.99, 1.44)
	3rd trimester	39,843	1,182	1.17 (0.98, 1.39)	1.19 (0.99, 1.42)	1.23* (1.02, 1.48)
**Local PM_{10}**	Entire pregnancy	36,736	1,095	1.00 (0.83, 1.19)	1.07 (0.89, 1.29)	1.18 (0.98, 1.42)
	1st trimester	39,647	1,180	0.97 (0.82, 1.15)	1.06 (0.89, 1.26)	1.15 (0.96, 1.38)
	2nd trimester	38,635	1,151	1.09 (0.92, 1.230)	1.00 (0.83, 1.20)	1.22* (1.01, 1.48)
	3rd trimester	39,280	1,165	1.10 (0.92, 1.32)	1.12 (0.94, 1.34)	1.25* (1.03, 1.51)

**Total $PM_{2.5}$**	Entire pregnancy	29,633	904	1.06 (0.86, 1.31)	1.13 (0.92, 1.40)	1.26 (1.00, 1.59)
	1st trimester	36,814	1,100	1.22* (1.02, 1.46)	1.11 (0.91, 1.34)	1.17 (0.96, 1.43)
	2nd trimester	33,811	1,023	1.05 (0.86, 1.28)	1.11 (0.90, 1.37)	1.09 (0.88, 1.36)
	3rd trimester	32,930	1,009	0.99 (0.82, 1.20)	0.94 (0.76, 1.14)	1.14 (0.93, 1.39)
**Total PM_{10}**	Entire pregnancy	36,744	1,097	0.98 (0.81, 1.18)	1.05 (0.86, 1.27)	1.28* (1.02, 1.59)
	1st trimester	39,663	1,182	1.10 (0.92, 1.31)	1.17 (0.97, 1.40)	1.14 (0.94, 1.39)
	2nd trimester	38,627	1,151	1.07 (0.90, 1.29)	1.04 (0.86, 1.25)	1.22* (1.00, 1.48)
	3rd trimester	39,280	1,165	1.05 (0.88, 1.26)	1.02 (0.85, 1.23)	1.09 (0.89, 1.32)
**NO_x**	Entire pregnancy	39,484	1,174	1.16 (0.98, 1.38)	1.17 (0.98, 1.40)	1.38** (1.15, 1.65)
	1st trimester	40,093	1,188	1.05 (0.89, 1.25)	1.15 (0.97, 1.37)	1.25* (1.05, 1.50)
	2nd trimester	40,544	1,206	1.11 (0.93, 1.31)	1.18 (1.00, 1.41)	1.32** (1.10, 1.58)
	3rd trimester	42,229	1,248	1.21* (1.02, 1.43)	1.12 (0.94, 1.33)	1.35** (1.13, 1.61)

§Adjusted for maternal age, body mass index, parity, smoking, diabetes mellitus, gestational diabetes, essential hypertension, gestational hypertension, maternal country of birth, education level, annual household income, fetal sex, year and season of birth. *p-value <0.05. **p-value <0.01.

Table S7. The characteristics of the participants by the clinical subtypes of preeclampsia: early- and late-onset preeclampsia.

Characteristics	Total, N (%)	PE, n (%)	Early-onset PE, n (%)	Late-onset PE, n (%)
Births				
	43,688 (100)	1,286 (2.9)	31 (0.1)	1,255 (2.9)
Maternal age				
≤19	758 (1.7)	23 (1.8)	0 (0)	23 (1.8)
20-34	34,610 (79.2)	1,000 (77.8)	24 (77.4)	976 (77.8)
≥35	8,320 (19.0)	263 (20.5)	7 (22.6)	256 (20.4)
Parity				
Nulliparous	21,294 (48.7)	895 (69.6)	20 (64.5)	875 (69.7)
Parous	22,394 (51.3)	391 (30.4)	11 (35.5)	380 (30.3)
Pre-pregnancy BMI				
<18.5	1,017 (2.3)	16 (1.2)	1 (3.2)	15 (1.2)
18.5-24.9	24,097 (55.2)	515 (40.0)	9 (55.2)	506 (40.3)
25-29.9	9,377 (21.5)	325 (25.3)	6 (19.4)	319 (25.4)
≥30	3,996 (9.1)	246 (19.1)	1 (3.2)	245 (19.5)
Missing	5,201 (11.9)	184 (14.3)	14 (45.2)	170 (13.5)
Smoking (cigarettes/day)				
Non-smoker	36,662 (83.9)	1,097 (85.3)	16 (51.6)	1,081 (86.1)
<10	2,854 (6.5)	55 (4.3)	2 (6.5)	53 (4.2)
≥10	1,145 (2.6)	22 (1.7)	0 (0)	22 (1.8)
Missing	3,027 (6.9)	112 (8.7)	13 (41.9)	99 (7.9)
Maternal health				
Diabetes Mellitus	298 (0.7)	27 (2.1)	2 (6.5)	25 (2.0)
Gestational Diabetes	1,203 (2.8)	76 (5.9)	5 (16.1)	71 (5.7)
Essential hypertension	204 (0.5)	38 (3.0)	1 (3.2)	37 (2.9)

Gestational hypertension	703 (1.6)	149 (11.6)	2 (6.5)	147 (11.7)
Maternal education				
Pre-secondary	5,369 (12.3)	139 (10.8)	4 (12.9)	135 (10.8)
Secondary	18,080 (41.4)	596 (46.3)	17 (54.8)	579 (46.1)
Post-secondary	18,451 (42.2)	517 (40.2)	10 (32.3)	507 (40.4)
Missing	1,788 (4.1)	34 (2.6)	0 (0)	34 (2.7)
Household income (SEK/year)				
<200,000	9,846 (22.5)	285 (22.2)	9 (29.0)	276 (22.0)
200,000–300,000	10,559 (24.2)	349 (27.1)	9 (29.0)	340 (27.1)
300,000–400,000	11,699 (26.8)	349 (27.1)	10 (32.3)	339 (27.0)
>400,000	10,540 (24.1)	274 (21.3)	3 (9.7)	271 (21.6)
Missing	1,044 (2.4)	29 (2.3)	0 (0)	29 (2.3)
Maternal country of birth				
Nordic country	31,172 (71.4)	985 (76.8)	23 (74.2)	962 (76.9)
Other country	12,457 (28.6)	297 (23.2)	8 (25.8)	289 (23.1)
Fetal sex				
Male	22,515 (51.5)	682 (53.0)	22 (71.0)	660 (52.6)
Female	21,173 (48.5)	604 (47.0)	9 (29.0)	595 (47.4)
Year of birth				
2000	3,820 (8.7)	98 (7.6)	8 (25.8)	90 (7.2)
2001	4,003 (9.2)	110 (8.6)	4 (12.9)	106 (8.4)
2002	4,486 (10.3)	129 (10.0)	6 (19.4)	123 (9.8)
2003	4,507 (10.3)	104 (8.1)	5 (16.1)	99 (7.9)
2004	4,578 (10.5)	120 (9.3)	5 (16.1)	115 (9.2)
2005	4,767 (10.9)	156 (12.1)	3 (9.7)	153 (12.2)
2006	4,188 (9.6)	169 (13.1)	0 (0)	169 (13.5)
2007	4,685 (10.7)	147 (11.4)	0 (0)	147 (11.7)
2008	4,723 (10.8)	140 (10.9)	0 (0)	140 (11.2)
2009	3,931 (9.0)	113 (8.8)	0 (0)	113 (9.0)
Season of birth				
Winter	10,298 (23.6)	306 (23.8)	4 (12.9)	302 (24.1)
Spring	11,245 (25.7)	363 (28.2)	8 (25.8)	355 (28.3)
Summer	11,121 (25.5)	281 (21.9)	11 (35.5)	270 (21.5)
Autumn	11,024 (25.2)	336 (26.1)	8 (25.8)	328 (26.1)

Table S8. Adjusted analysis for PE with SGA vs. PE without SGA in relation to the exposure quartiles of black carbon particles (μg/m³) during each window of exposure.

	Black carbon **Adjusted OR§ (95% CI)**	
Range, μg/m³	**PE with SGA**	**PE without SGA**
**Entire pregnancy**	201/31607	734/29073
**Linear (1 μg/m³)**	3.48 (1.67, 7.27)**	1.89 (1.24, 2.88)**
**Q1 (0.03 – 0.21 μg/m³)**	Reference	Reference
**Q2 (0.21 – 0.36 μg/m³)**	1.27 (0.82, 1.98)	1.16 (0.93, 1.45)
**Q3 (0.36 – 0.48 μg/m³)**	1.26 (0.80, 1.97)	1.38 (1.10, 1.72)**
**Q4 (0.48 – 1.93 μg/m³)**	1.74 (1.13, 2.67)*	1.36 (1.08, 1.70)**
**First trimester**	209/32783	761/30148
**Linear (1 μg/m³)**	2.94 (1.53, 5.63)**	1.67 (1.13, 2.46)*
**Q1 (0.03 – 0.23 μg/m³)**	Reference	Reference
**Q2 (0.23 – 0.37 μg/m³)**	1.09 (0.71, 1.68)	1.04 (0.83, 1.31)
**Q3 (0.37 – 0.49 μg/m³)**	1.23 (0.81, 1.86)	1.34 (1.08, 1.67)**
**Q4 (0.49 – 2.94 μg/m³)**	1.52 (1.00, 2.32)	1.31 (1.04, 1.64)*
**Second trimester**	206/32705	770/30083
**Linear (1 μg/m³)**	3.20 (1.59, 6.42)**	1.87 (1.24, 2.81)**
**Q1 (0.02 – 0.18 μg/m³)**	Reference	Reference

**Q2 (0.18 – 0.30 μg/m^3)**	1.43 (0.93, 2.21)	1.16 (0.93, 1.43)
**Q3 (0.30 – 0.42 μg/m^3)**	1.34 (0.85, 2.10)	1.17 (0.93, 1.46)
**Q4 (0.42 – 2.01 μg/m^3)**	1.82 (1.18, 2.83)**	1.45 (1.17, 1.82)**
**Third trimester**	207/32831	775/30185
**Linear (1 μg/m^3)**	2.60 (1.33, 5.07)**	1.64 (1.13, 2.37)**
**Q1 (0.03 – 0.21 μg/m^3)**	Reference	Reference
**Q2 (0.21 – 0.37 μg/m^3)**	1.20 (0.77, 1.85)	1.05 (0.84, 1.30)
**Q3 (0.37 – 0.52 μg/m^3)**	1.41 (0.91, 2.18)	1.19 (0.95, 1.49)
**Q4 (0.52 – 2.31 μg/m^3)**	1.71 (1.12, 2.60)*	1.28 (1.03, 1.59)*

§Adjusted for maternal age, body mass index, parity, smoking, diabetes mellitus, gestational diabetes, essential hypertension, gestational hypertension, maternal country of birth, education level, annual household income, fetal sex, year and season of birth. *p-value <0.05 as compared to the healthy controls. **p-value <0.01 as compared to the healthy controls.

Table S9. Adjusted analyses for PE with SGA vs. PE without SGA in relation to the exposure quartiles of local and total PM$_{2.5}$ (μg/m^3) during each window of exposure.

Local PM$_{2.5}$			Total PM$_{2.5}$		
Adjusted OR§ (95% CI)			Adjusted OR§ (95% CI)		
**Range, μg/m^3**	**PE with SGA**	**PE without SGA**	**Range, μg/m^3**	**PE with SGA**	**PE without SGA**
Entire pregnancy	**193/30,192**	**700/27,747**		**154/24,462**	**588/22,462**
**Linear (5 μg/m^3)**	4.33 (1.56, 12.04)**	2.56 (1.48, 4.43)**		1.49 (0.56, 3.94)	2.20 (1.34, 3.61)**
**Q1 (0.13 – 0.96)**	Reference	Reference	Q1 (6.85 – 10.34)	Reference	Reference
**Q2 (0.96 – 1.50)**	1.09 (0.69, 1.73)	1.18 (0.94, 1.49)	Q2 (10.34 – 11.02)	0.98 (0.60, 1.60)	1.07 (0.82, 1.39)
**Q3 (1.50 – 2.08)**	1.42 (0.92, 2.21)	1.24 (0.98, 1.57)	Q3 (11.02 – 11.76)	1.06 (0.65, 1.73)	1.32 (1.02, 1.72)*
**Q4 (2.08 – 7.52)**	1.65 (1.06, 2.58)*	1.50 (1.19, 1.90)**	Q4 (11.76 – 17.30)	1.05 (0.60, 1.83)	1.54 (1.15, 2.05)**
First trimester	208/32,332	754/29,726		196/30,390	713/27,934
**Linear (5 μg/m^3)**	2.57 (1.13, 5.82)*	1.96 (1.24, 3.10)**		1.45 (0.95, 2.23)	1.30 (1.03, 1.65)*
**Q1 (0.09 – 0.89)**	Reference	Reference	Q1 (6.19 – 9.64)	Reference	Reference
**Q2 (0.89 – 1.42)**	1.19 (0.77, 1.83)	1.01 (0.81, 1.25)	Q2 (9.64 – 10.94)	1.51 (0.96, 2.37)	1.18 (0.95, 1.47)
**Q3 (1.42 – 2.11)**	1.36 (0.89, 2.09)	1.21 (0.97, 1.50)	Q3 (10.94 – 12.12)	1.72 (1.09, 2.71)*	1.07 (0.84, 1.36)
**Q4 (2.11 – 10.68)**	1.59 (1.03, 2.45)*	1.32 (1.05, 1.65)*	Q4 (12.12 – 22.06)	1.51 (0.92, 2.48)	1.22 (0.95, 1.56)
Second trimester	199/31,800	747/29,230		174/27,798	670/25,546
**Linear (5 μg/m^3)**	2.87 (1.26, 6.55)*	2.00 (1.28, 3.11)**		1.36 (0.86, 2.15)	1.10 (0.86, 1.40)
**Q1 (0.08 – 0.88)**	Reference	Reference	Q1 (6.23 – 9.63)	Reference	Reference
**Q2 (0.88 – 1.40)**	0.77 (0.49, 1.21)	1.03 (0.82, 1.28)	Q2 (9.63 – 10.89)	1.23 (0.75, 2.02)	1.01 (0.79, 1.30)
**Q3 (1.40 – 2.09)**	1.20 (0.80 1.81)	1.02 (0.82, 1.28)	Q3 (10.89 – 12.11)	1.19 (0.71, 2.01)	1.09 (0.84, 1.41)
**Q4 (2.09 – 10.5)**	1.29 (0.84, 1.99)	1.31 (1.04, 1.65)*	Q4 (12.11 – 21.31)	1.37 (0.81, 2.32)	1.09 (0.83, 1.42)
Third trimester	206/32,800	773/30,149		167/26,999	660/24,799
**Linear (5 μg/m^3)**	2.65 (1.19, 5.92)*	1.65 (1.07, 2.53)*		0.92 (0.58, 1.44)	1.37 (1.09, 1.72)**
**Q1 (0.10 – 0.83)**	Reference	Reference	Q1 (5.44 – 9.72)	Reference	Reference
**Q2 (0.83 – 1.35)**	1.71 (1.10, 2.65)*	1.20 (0.97, 1.49)	Q2 (9.72 – 10.94)	1.13 (0.73, 1.77)	1.00 (0.78, 1.28)
**Q3 (1.35 – 2.05)**	1.72 (1.10, 2.68)*	1.26 (1.01, 1.57)*	Q3 (10.94 – 12.18)	0.92 (0.57, 1.48)	1.07 (0.83, 1.38)
**Q4 (2.05 – 9.64)**	1.92 (1.21, 3.05)**	1.25 (0.99, 1.58)	Q4 (12.18 – 25.75)	0.83 (0.51, 1.38)	1.40 (1.09, 1.80)**

§Adjusted for maternal age, body mass index, parity, smoking, diabetes mellitus, gestational diabetes, essential hypertension, gestational hypertension, maternal country of birth, education level, annual household income, fetal sex, year and season of birth. *p-value <0.05 as compared to the healthy controls. **p-value <0.01 as compared to the healthy controls.**Table S10.** Adjusted analyses for PE with SGA vs. PE without SGA in relation to the exposure quartiles of local and total PM$_{10}$ (μg/m^3) during each window of exposure.

**Local PM$_{10}$** Adjusted OR§ (95% CI)			**Total PM$_{10}$** Adjusted OR§ (95% CI)		
**Range, μg/m^3**	**PE with SGA**	**PE without SGA**	**Range, μg/m^3**	**PE with SGA**	**PE without SGA**
**Entire pregnancy**	198/30,323	710/27,896		198/30,328	711/27,902
Linear (5 μg/m^3)	1.73 (1.06, 2.82)*	1.48 (1.14, 1.92)**		1.77 (1.14, 2.75)*	1.34 (1.06, 1.69)*

Q1 (0.20 – 1.60)	Reference	Reference	Q1 (10.25 – 14.12)	Reference	Reference
Q2 (1.60 – 2.76)	1.01 (0.65, 1.58)	1.14 (0.91, 1.43)	Q2 (14.12 – 15.64)	2.33 (1.47, 3.69)**	0.84 (0.66, 1.06)
Q3 (2.76- 4.03)	1.22 (0.80, 1.88)	1.24 (0.98, 1.56)	Q3 (15.64 – 17.27)	2.25 (1.38, 3.67)**	1.00 (0.79, 1.27)
Q4 (4.03 – 9.97)	1.48 (0.96, 2.27)	1.39 (1.10, 1.76)**	Q4 (17.27 – 25.62)	2.27 (1.29, 3.98)**	1.33 (1.01, 1.73)*
**First trimester**	208/32,635	765/30,011		208/32,645	766/30,021
Linear (5 μg/m³)	1.46 (1.01, 2.12)*	1.30 (1.06, 1.60)*		1.40 (1.08, 1.82)*	1.19 (1.03, 1.38)*
Q1 (0.12 – 1.42)	Reference	Reference	Q1 (8.58 – 13.70)	Reference	Reference
Q2 (1.42 – 2.44)	0.95 (0.62, 1.47)	1.03 (0.83, 1.28)	Q2 (13.70 – 15.61)	1.30 (0.84, 2.03)	1.21 (0.97, 1.50)
Q3 (2.44 – 4.13)	1.31 (0.86, 1.98)	1.18 (0.95, 1.46)	Q3 (15.61 – 17.54)	1.94 (1.27, 2.97)**	1.22 (0.97, 1.53)
Q4 (4.13 – 13.74)	1.44 (0.93, 2.22)	1.32 (1.04, 1.66)*	Q4 (17.54 – 36.24)	1.45 (0.90, 2.33)	1.20 (0.94, 1.53)
**Second trimester**	204/31,860	750/29,299		204/31,855	750/29,295
Linear (5 μg/m³)	1.41 (0.97, 2.06)	1.32 (1.08, 1.61)**		1.19 (0.91, 1.56)	1.09 (0.94, 1.26)
Q1 (0,12 – 1.41)	Reference	Reference	Q1 (8.7 – 13.68)	Reference	Reference
Q2 (1.41 – 2.37)	0.98 (0.64, 1.49)	1.07 (0.86, 1.34)	Q2 (13.68 – 15.51)	1.01 (0.64, 1.58)	1.15 (0.92, 1.43)
Q3 (2.37 – 4.11)	1.14 (0.75, 1.74)	1.07 (0.85, 1.34)	Q3 (15.51 – 17.61))	1.33 (0.86, 2.05)	1.15 (0.91, 1.44)
Q4 (4.11 – 14.17	1.29 (0.83, 2.01)	1.34 (1.06, 1.69)*	Q4 (17.61 – 36.19)	1.61 (1.02, 2.55)*	1.22 (0.95, 1.55)
**Third trimester**	206/32,474	766/29,862		206/32,474	766/29,862
Linear (5 μg/m³)	1.44 (0.99, 2.11)	1.30 (1.07, 1.58)**		1.09 (0.85, 1.40)	1.12 (0.98, 1.28)
Q1 (0.10 – 1.28)	Reference	Reference	Q1 (6.58 – 13.29)	Reference	Reference
Q2 (1.28 – 2.26)	1.93 (1.24, 2.99)**	1.09 (0.87, 1.36)	Q2 (13.29 – 15.38)	1.11 (0.72, 1.69)	1.01 (0.81, 1.26)
Q3 (2.26 – 3.94)	1.62 (1.02, 2.57)*	1.18 (0.95, 1.48)	Q3 (15.38 – 17.51)	1.35 (0.88, 2.06)	1.01 (0.80, 1.27)
Q4 (3.94 – 13.19)	2.20 (1.37, 3.54)**	1.30 (1.02, 1.64)*	Q4 (17.51 – 38.36)	1.03 (0.64, 1.66)	1.26 (0.99, 1.60)

§Adjusted for maternal age, body mass index, parity, smoking, diabetes mellitus, gestational diabetes, essential hypertension, gestational hypertension, maternal country of birth, education level, annual household income, fetal sex, year and season of birth. *p-value <0.05 as compared to the healthy controls. **p-value <0.01 as compared to the healthy controls.
